# Comparison of three nutritional screening tools for detecting sarcopenia in patients with maintenance hemodialysis

**DOI:** 10.3389/fpubh.2022.996447

**Published:** 2022-10-24

**Authors:** Xiaoyu Chen, Peipei Han, Xiaoyan Zhu, Peiyu Song, Yinjiao Zhao, Hui Zhang, Chen Yu, Jianying Niu, Wei Ding, Junli Zhao, Liming Zhang, Hualin Qi, Suhua Zhang, Qi Guo

**Affiliations:** ^1^Department of Rehabilitation Medicine, Shanghai University of Medicine and Health Sciences Affiliated Zhoupu Hospital, Shanghai, China; ^2^Department of Rehabilitation Medicine, Shanghai Herson Rehabilitation Hospital, Shanghai, China; ^3^Jiangwan Hospital of Shanghai Hongkou District, Shanghai University of Medicine and Health Sciences Affiliated First Rehabilitation Hospital, Shanghai, China; ^4^Department of Nephrology, Tongji Hospital, School of Medicine, Tongji University, Shanghai, China; ^5^Department of Nephrology, The Fifth People's Hospital of Shanghai, Fudan University, Shanghai, China; ^6^Department of Nephrology, Shanghai Ninth People's Hospital, Shanghai Jiao Tong University School of Medicine, Shanghai, China; ^7^Department of Nephrology, Shanghai University of Medicine and Health Science Affiliated Zhoupu Hospital, Shanghai, China; ^8^Department of Nephrology, Zhabei Central Hospital of Jing'an District of Shanghai, Shanghai, China; ^9^Department of Nephrology, Shanghai Pudong New Area People's Hospital, Shanghai, China; ^10^Department of Nephrology, Suzhou Kowloon Hospital, Shanghai Jiao Tong University School of Medicine, Suzhou, China

**Keywords:** dynapenia, geriatric nutritional risk index, hemodialysis, nutritional screening tool, pre-sarcopenia, sarcopenia

## Abstract

**Background:**

Malnutrition, dynapenia, and sarcopenia are prevalent conditions among patients with maintenance hemodialysis (MHD). They are related to numerous adverse health outcomes. The aim of this study was to compare the effect of three nutritional screening tools on predicting the risk of dynapenia and sarcopenia in patients with MHD.

**Methods:**

From July 2020 to April 2021, a total of 849 patients with MHD were enrolled at seven different healthcare facilities in Shanghai, China in this multi-center cross-sectional study. Geriatric nutritional risk index (GNRI), malnutrition inflammation score (MIS), and creatinine (Cr) index were used for nutritional assessment. The cutoff values of muscle mass and strength to define dynapenia, pre-sarcopenia, and sarcopenia were based on the consensus by the Asia Working Group of Sarcopenia in 2019.

**Results:**

Among 849, almost 60% were malnourished with the majority suffering from dynapenia (27.7%), followed by sarcopenia (22.7%), and pre-sarcopenia (6.2%).The area under the receiver–operating characteristic curve for GNRI was 0.722 [95% confidence interval (CI) = 0.684–0.760] and 0.723 (95% CI = 0.663–0.783) in predicting sarcopenia and pre-sarcopenia. The GNRI [odds ratio (OR) =6.28, 95% CI: 4.05–9.73], MIS (OR =1.91, 95% CI: 1.31–2.78), and the Cr index (OR =2.73, 95% CI: 1.71–4.34) were all significantly associated with the risk of sarcopenia. More importantly, the sarcopenia predictability of the GNRI appears greater than the MIS and Cr index, while MIS was similar to the Cr index. Similarly, the superiority of GNRI prediction was also found in pre-sarcopenia, but not in dynapenia.

**Conclusion:**

All the three nutritional screening tools were significantly associated with an increased risk of sarcopenia. The sarcopenia predictability of the GNRI was greater than the MIS and Cr index.

## Introduction

Sarcopenia is a clinical condition characterized by an aged-related decrease in skeletal muscle mass and low muscle strength and/or physical performance (1). Muscle mass and muscle strength have been discussed together since 2010 when the European Working Group on Sarcopenia in Older People (EWGSOP) defined pre-sarcopenia and sarcopenia (2). Evidence is accumulating that both low muscle quantity and quality might relate to adverse clinical outcomes, including increased hospitalization, poorer quality of life, and increased mortality in patients with maintenance hemodialysis (MHD) (3–5). Interestingly, however, several recent studies suggest that the loss of muscle strength without low muscle mass as dynapenia, is an important risk for mortality in patients with MHD (6). Hence, there is a great interest in correctly differentiating the loss of muscle mass from strength. In fact, this deviation between the association of muscle mass with muscle strength and clinical adverse outcomes is a matter of interest and debate in the international scientific community (7, 8).

The patients with MHD are related to a range of causes of muscle mass and function, such as decreased physical activity and nutrition intake, hormone dysfunction, and chronic inflammation (9). Among these, malnutrition is a significant risk factor for the development of sarcopenia (10). A wide variety of nutritional screening markers and tools are available to assess the nutritional status of patients with MHD (11), such as body mass index (BMI), serum creatinine (Cr), and serum albumin. These markers are insufficient when used alone, so many clinicians usually use them in combination in clinical practice. The malnutrition–inflammation score (MIS) (12) is a valid diagnostic tool for evaluating nutritional status in patients with MHD. However, the MIS is time-consuming and cumbersome, because it requires a subjective evaluation. Indeed, several simple and completely objective nutritional screening tools can also be used to evaluate the nutritional risk of patients with MHD. The Cr index and geriatric nutritional risk index (GNRI) are recommended as the simple risk indexes for nutritional status assessment among patients with MHD (13). These two markers are objective and do not need special skills or experience but instead can easily calculated from the results of routine blood tests obtained at the bedside. Previous reports have reported the association between the nutritional marks and sarcopenia among the hospitalized older adults (14) and kidney transplant recipients (15). The conclusions are inconsistent as different nutritional indicators were used. To date, there are no research on the association between dynapenia and nutritional status in the dialysis population, so further study is needed.

In addition, few studies have compared the usefulness and predictive ability of these three nutritional indexes (MIS, GNRI, and Cr index) regarding dynapenia, pre-sarcopenia and sarcopenia in this population. It is clinically important to directly compare these three indexes in the hemodialysis population comprising a relatively large number of patients with MHD. Thus, the aim of this study was to evaluate the ability of the three nutritional screening tools (MIS, GNRI, and Cr index) to identify dynapenia, pre-sarcopenia, and sarcopenia in patients with MHD.

## Methods

### Subjects

**Figure 1:**
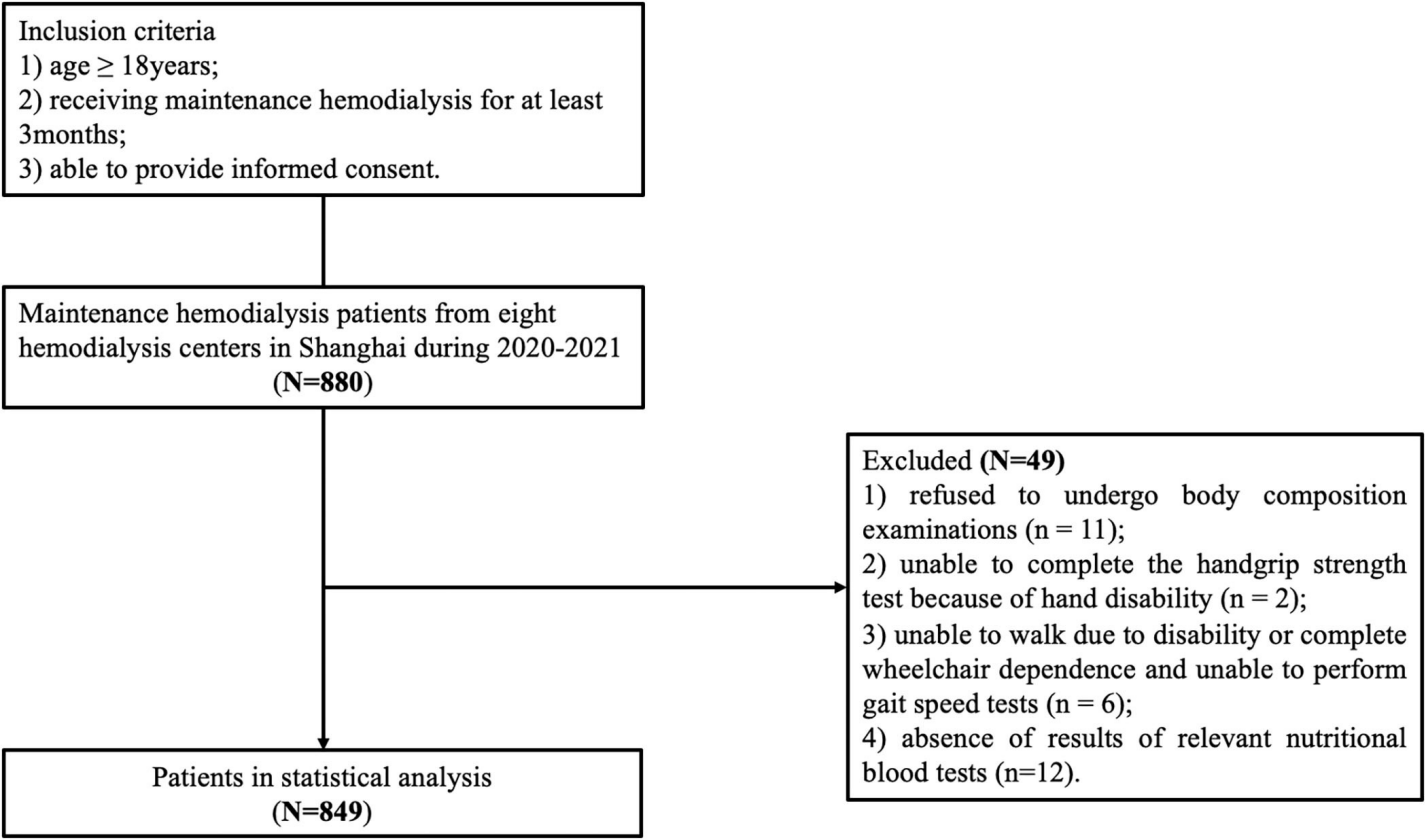
Flow chart of the study.

A multi-center cross-sectional study was conducted in Shanghai between July 2020 and April 2021and included seven hemodialysis centers. The inclusion were (1) age ≥ 18 years, (2) receiving maintenance hemodialysis for at least 3 months, (3) able to provide informed consent, while the exclusion criteria included (1) missing data on diagnostic criteria for sarcopenia, (2) did not complete date on the nutrition assessments, (3) clinical instability (presented with an infection, pulmonary edema, amputated limb, or malignancy), and (4) unable to communicate with the researchers or refusal to participate in this study. We excluded patients who: (1) refused to undergo body composition examinations (*n* = 11); (2) unable to complete the handgrip strength test because of hand disability (*n* = 2); (3) unable to walk due to disability or complete wheelchair dependence and unable to perform gait speed tests (*n* = 6); and 4) absence of results of relevant nutritional blood tests (*n* = 12). The remaining 849 patients were included in the final analytic sample (Figure 1). The Ethics Committee of Shanghai University of Medicine and Health Sciences approved this study (number 2019-A4-2621-19-201001-03-12010419771113601X), and all of the patients provided written informed consent to take part in this study. These methods were implemented in accordance with the principles of the Declaration of Helsinki.

### Covariates

All the patients were invited to a face-to-face interview to answer a standardized questionnaire. Covariates included socio-demographic characteristics and chronic disease status. Demographic characteristics comprised of age, sex, post-dialysis weight, dialysis vintage, and education level. The International Physical Activity Questionnaire (IPAQ) (16) was used to evaluate the physical activity. Comorbidity was assessed using the Charlson Comorbidity Index (CCI), which accounted for multiple comorbidity by creating a summation score based on 19 comorbidity conditions (17). Moreover, all the blood samples were taken prior to dialysis. The dialysis quality was assessed by fractional clearance index for urea (Kt/V), which was monitored in every dialysis treatment using the OCM^®^ (On-line Clearance Monitor; Fresenius Medical Care, Bad Homburg, Germany).

### Definition of dynapenia, pre-sarcopenia, and sarcopenia

A direct segmental multi-frequency bioelectrical impedance analysis (BIA; InBody S10; Biospace, Seoul, Korea) was used to measure muscle mass (18). Low muscle mass was defined as skeletal muscle index (SMI) lower than 7.0 and 5.7 kg/m^2^ in man and women, respectively. The dynamometer (GRIP-D; Takei Ltd, Niigata, Japan) on the non-fistula hand before a dialysis session to evaluate the muscle strength.

For patients with an indwelling dialysis catheter, we used the dominant hand to test muscle strength. Low muscle strength was defined as handgrip strength < 28 or < 18 kg in males and females. Usual gait speed was measured to assess poor-physical performance, which was less than 1.0 m/s in both males and females. All the patients were measured for these tests using the same measurement by a physical therapist at each facility on dialysis day before dialysis treatment.

Dynapenia was defined as patients with decreased muscle strength and normal muscle mass (8). A low SMI with normal handgrip strength and normal gait speed were to defined pre-sarcopenia (2). Sarcopenia was diagnosed based on 2019 new version of the Asian Working Group for Sarcopenia (AWGS) criteria. These criteria included both low muscle mass and low muscle strength and/or poor-physical performance (1).

### Assessment of nutritional status

#### Malnutrition–inflammation score

The Malnutrition–Inflammation Score (MIS) is a comprehensive scoring system that is associated with expected hospitalization rates, mortality rates, and nutritional measures of patients with MHD (12). The 10 items about the patient's with MHD nutritional and functional state were included in the MIS. Each component of the MIS has four severity levels, ranging from 0 (normal) to 3 (severely abnormal). The total score ranges from 0 to 30. The higher score suggests a more severe degree of malnutrition status.

#### Geriatric nutritional risk index

In the several previous studies, Geriatric Nutritional Risk Index (GNRI) has been shown to be an important predictor of morbidity and mortality and associated with a variety of nutrition-related markers (19, 20). The GNRI was calculated using the following equation (21):


$$\begin{array}{l} \text{GNRI}=\left[14.89\times \text{albumin}\left(\text{g}/\text{dl}\right)\right]\\ +\left[41.7\times \left(\text{bodyweight}/\text{idealbody weight}\right)\right] \end{array}$$

Ideal body weight was defined as having a body mass index (BMI) value of 22 kg/m^2^.

#### Creatinine index

Creatinine (Cr) index was calculated based on known sex differences as follows (13, 22):


$$\begin{array}{l} \text{Crindex for men}=16.21+1.12-0.06\times \left[\text{age }\left(\text{year}\right)\right]\\ -0.08\;\times \left(\text{single pool Kt}/\text{V}\right)+0.009\\ \times \left[\text{serum creatinine }\left(\text{μmol}/\text{L}\right)\right],\\ \text{Cr index for women}=16.21-0.06\times \left[\text{age }\left(\text{year}\right)\right]\\ -0.08\;\times \left(\text{single pool Kt}/\text{V}\right)+0.009\\ \times \left[\text{serum creatinine }\left(\text{μmol}/\text{L}\right)\right]. \end{array}$$

## Statistics

Independent *t*-test (numeric variables) or by chi-square test (categorical variables) were used to compared patients with and without sarcopenia. Numeric variables with a normal distribution are expressed as the mean ± SD, while with a non-normal distribution are expressed as the median, with the 25–75% interquartile ranges given in parentheses. Categorical variables used an absolute number and proportions. The receiver operating characteristic curve (ROC) was a graph of sensitivity plotted against (1–specificity) overall possible diagnostic cut points. The cutoff points of dynapenia, pre-sarcopenia and sarcopenia were determined by the maximal Youden's index, which was calculated as (sensitivity + specificity −1) and the greatest combination of sensitivity and specificity. Logistic regression analyses were used to assess the relationships between three nutritional screening tools (MIS, GNRI, and Cr index) and dynapenia, pre-sarcopenia, or sarcopenia. Adjusted model included age, sex, Kt/V, IPAQ, CCI, Cr, PTH, and phosphorus. We further used Harrell's C-statistics to confirm which nutritional markers were best for identifying dynapenia, pre-sarcopenia, and sarcopenia. *P* < 0.05 was considered statistically significant. IBM SPSS Statistics v26.0 (SPSS Inc., Chicago, IL, United States) was used to perform all the statistical analyses.

## Results

### Characteristics of the patients for sarcopenia

Among 849 participants (520 men, 329 women; mean age 61.4 ±12.6 years), and there were 235 (27.7%) patients with dynapenia, followed by 193 (22.7%) patients with sarcopenia, and 53 (6.2%) with pre-sarcopenia, respectively. Table 1 shows the socioeconomic and health-related characteristics of patients with MHD stratified by sarcopenic status. Age, post-dialysis weight, BMI, Kt/v, handgrip strength, SMI, gait speed, IPAQ, nutritional factors (MIS, GNRI, and Cr index), CCI, and laboratory parameters (albumin, Cr, PTH, and phosphorus) significantly differed between groups (*P* < 0.05, Table 1).

**Table 1:** Baseline characteristics of study participants according to the presence of sarcopenia.

**Characteristics**	**Non-sarcopenia**	**Sarcopenia**	
	**(*n* = 656)**	**(*n* = 193)**	***P-*value**
Age (y)	59.4 ± 12.3	68.3 ± 11.3	< 0.001
Male (%)	404(61.6)	116(60.1)	0.710
Post-dialysis weight (kg)	65.2 ± 12.0	53.5 ± 7.9	< 0.001
BMI (kg/m^2^)	24.0 ± 3.8	21.1 ± 2.9	< 0.001
Vintage (months)	46.2(22.8,91.4)	48.2(30.1,105.3)	0.062
Kt/v	1.33 ± 0.33	1.49 ± 0.30	< 0.001
Handgrip strength	26.6 ± 8.7	18.8 ± 6.2	< 0.001
SMI (kg/m^2^)	7.3 ± 1.1	5.8 ± 0.8	< 0.001
Gait speed (m/s)	1.04 ± 0.28	0.78 ± 0.31	< 0.001
IPAQ (Met-min/wk)	1,508(693.3492)	783(0,2079)	0.001
**Nutritional factors**			
GNRI	104.0 ± 9.8	97.8 ± 7.2	< 0.001
MIS	3.9 ± 2.8	5.4 ± 3.3	< 0.001
Cr index	22.4 ± 3.0	20.4 ± 2.5	< 0.001
Number of medications (n)	4.4 ± 2.4	4.5 ± 2.5	0.709
CCI	3.8 ± 1.6	4.2 ± 1.8	0.001
**Laboratory parameters**			
Hemoglobin (g/dL)	110.9 ± 15.7	111.3 ± 16.3	0.720
Albumin (g/L)	39.8 ± 3.5	38.8 ± 3.4	0.001
Cr (μmol/L)	1013.5 ± 276.6	864.1 ± 223.5	< 0.001
PTH (pg/dL)	375.2 ± 332.7	303.4.3 ± 288.0	0.007
Calcium (mg/dL)	2.27 ± 0.25	2.25 ± 0.27	0.180
Phosphorus (mg/dL)	1.99 ± 0.63	1.84 ± 0.65	0.004

BMI, body mass index; Kt/V, fractional clearance index for urea; ESRD, end stage renal disease; SMI, skeletal muscle index; GNRI, geriatric nutritional risk index; MIS, malnutrition inflammation score; Cr, creatinine; IPAQ, international physical activity questionnaire; Met-min/wk, metabolic equivalent task minutes per week; CCI, Charlson comorbidity index; PTH, parathyroid hormone.

### Evaluations of three nutritional screening tools on ROC curve

**Figure 2:**
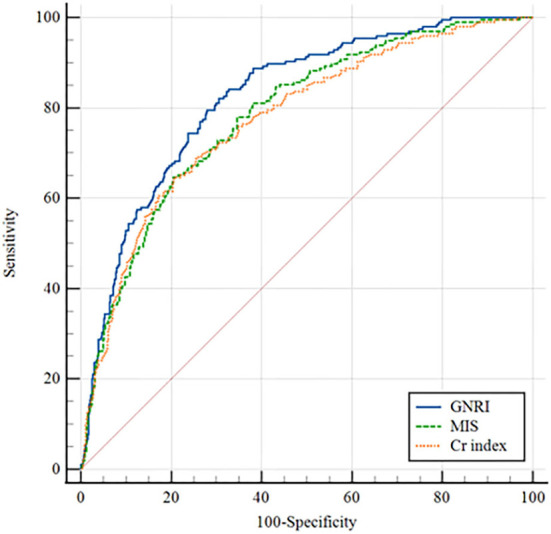
Receiver operating characteristic (ROC) curve for geriatric nutritional risk index (GNRI), malnutrition inflammation score (MIS), and Creatinine (Cr) index according to sarcopenia.

The ROC curves were created to quantify sensitivity, specificity, areas under the ROC curves (AUC), and optimal cutoff points of three nutritional screening tools. Table 2 shows that the AUC of GNRI toward sarcopenia and pre-sarcopenia were lager than Cr index and MIS. The AUC of MIS, GNRI, and Cr index toward sarcopenia were 0.640, 0.722, and 0.700, respectively (Figure 2), while pre-sarcopenia was 0.517, 0.723, and 0.558, respectively. Meanwhile, the cutoff points of GNRI toward pre-sarcopenia and sarcopenia were 103 and 102.4, respectively.

**Table 2:** Receiver operating characteristic curve for the nutritional factors to estimate the probability of dynapenia, pre-sarcopenia, and sarcopenia.

**Variables**	**Bootstrap ROC curve**				
	**AUC (95%CI)**	***P-*value**	**Cutoff**	**Sensitivity (%)**	**Specificity (%)**
**Dynapenia**					
MIS	0.582(0.535,0.628)	< 0.001	5.5	33.6	82.6
GNRI	0.471(0.425,0.517)	0.208	94.4	14.9	90.2
Cr index	0.606(0.562,0.651)	< 0.001	22.5	67.2	50.8
**Pre-sarcopenia**					
MIS	0.517(0.443,0.592)	0.674	3.5	56.6	52.9
GNRI	0.723(0.663,0.783)	< 0.001	102.4	77.4	63.2
Cr index	0.558(0.490,0.626)	0.161	24.1	92.5	28.4
**Sarcopenia**					
MIS	0.640(0.596,0.684)	< 0.001	4.5	54.4	66.2
GNRI	0.722(0.684,0.760)	< 0.001	103.0	79.3	56.6
Cr index	0.700(0.660,0.740)	< 0.001	21.1	67.4	66.5

ROC, receiver operating characteristic curve; AUC, area under the curve; CI, confidence interval; GNRI, geriatric nutritional risk index; MIS, malnutrition inflammation score; Cr, creatinine.

### Relationships of the three nutritional screening tools and dynapenia, pre-sarcopenia, and sarcopenia

The relationships between the three nutritional screening tools and dynapenia, pre-sarcopenia, and sarcopenia are shown in Table 3. After adjustment for age, sex, Kt/V, IPAQ, CCI, Cr, PTH, and phosphorus, a significantly higher risk of sarcopenia was found in patients in poor-nutrition categories identified by GNRI [odds ratio (OR) = 6.28, 95%CI: 4.05, 9.73) than Cr index (OR = 2.73, 95%CI: 1.71, 4.34) and MIS (OR = 1.91, 95%CI: 1.31, 2.78). The OR for pre-sarcopenia was higher in Cr index in pre-sarcopenia (OR = 5.49, 95% CI: 1.88, 16.02) than GNRI (OR = 5.29, 95% CI: 2.69, 10.39). However, the OR for dynapenia was slightly higher in MIS (OR = 2.12, 95% CI: 1.42, 3.17) than GNRI (OR = 1.71, 95% CI: 1.02, 2.86), and Cr index (OR = 1.73, 95% CI: 1.09, 2.73).

**Table 3:** Multivariable logistic regression analysis evaluating nutritional factors associated with dynapenia, pre-sarcopenia, and sarcopenia.

**Variables**	**OR (95%CI)**			
	**Crude**	***P***	**Adjusted model**	***P***
**Dynapenia**				
MIS	2.41(1.67,3.47)	< 0.001	2.12(1.42,3.17)	< 0.001
GNRI	1.53(0.96,2.46)	0.075	1.71(1.02,2.86)	0.040
Cr index	2.06(1.48,2.85)	< 0.001	1.73(1.09,2.73)	0.019
**Pre-sarcopenia**				
MIS	1.47 (0.83,2.58)	0.186	1.57(0.87,2.83)	0.132
GNRI	5.82(3.00,11.31)	< 0.001	5.29(2.69,10.39)	< 0.001
Cr index	3.86(1.51,9.87)	0.005	5.49(1.88,16.02)	0.002
**Sarcopenia**				
MIS	2.33(1.68,3.23)	< 0.001	1.91(1.31,2.78)	0.001
GNRI	4.86(3.33,7.09)	< 0.001	6.28(4.05,9.73)	< 0.001
Cr index	3.98(2.84,5.60)	< 0.001	2.73(1.71,4.34)	< 0.001

Adjusted model: Adjusted for age, sex, Kt/V, IPAQ, CCI, Cr, PTH and phosphorus. GNRI, geriatric nutritional risk index; MIS, malnutrition inflammation score; Cr, creatinine; Kt/V, fractional clearance index for urea; IPAQ, international physical activity questionnaire; CCI, Charlson comorbidity index; PTH, parathyroid hormone.

### Discrimination tests

Harrell's C statistic were used to evaluate the discrimination of each nutritional screening tool. The Harrell's C statistics of the GNRI and Cr index were significantly higher than the MIS (GNRI vs. MIS: *P* = 0.001; GNRI vs. Cr index: *P* = 0.001), and there was no significant difference between the MIS and the Cr index in the values of the Harrell's C statistic for sarcopenia (*P* = 0.436, Table 4). Pre-sarcopenia also showed the similar results; however, there were no significant difference among the three nutritional screening tools in the values of the Harrell's C statistic for dynapenia (Table 4).

**Table 4:** Results of the Harrell's C statistic.

**Variables**	**Harrell's C statistic (95%CI)**	**SE**	***P***	
**Dynapenia**				
MIS	0.713(0.677,0.747)	0.021	Ref.	0.267
GNRI	0.705(0.664,0.746)	0.021	0.267	Ref.
Cr index	0.703(0.667,0.737)	0.021	0.322	0.982
**Pre-sarcopenia**				
MIS	0.662(0.624,0.698)	0.040	Ref.	0.004
GNRI	0.757(0.722,0.790)	0.030	0.004	Ref.
Cr index	0.701(0.664,0.736)	0.034	0.184	0.120
**Sarcopenia**				
MIS	0.785(0.756,0.813)	0.018	Ref.	0.001
GNRI	0.828(0.801,0.853)	0.016	0.001	Ref.
Cr index	0.779(0.749,0.806)	0.019	0.436	0.001

GNRI, geriatric nutritional risk index; MIS, malnutrition inflammation score; Cr, creatinine.

## Discussion

Our study suggested that each of the three nutritional screening tools was significantly associated with an increased risk of dynapenia, pre-sarcopenia, and sarcopenia in Chinese patients with MHD. Moreover, the sarcopenia predictability of the GNRI appears greater than Cr index and MIS, while Cr index similar to the MIS. Thus, GNRI is considered a useful tool for predicting sarcopenia in this group.

In our study, the prevalence of sarcopenia was 22.7%, which is consistent with the previously reported sarcopenia prevalence in dialysis patients (20.0%) (6), as well as the results of a recent systematic analysis of 30 studies in dialysis patients (sarcopenia prevalence = 28.5%) (23). Based on the cut-off values of muscle mass and function recommended by existing guidelines for the diagnosis of sarcopenia in the general population, the prevalence of sarcopenia in hemodialysis patients ranges from 4 to 68% (6, 23–25). This wide range is partly representative of the lack of recognized definition for diagnosing low muscle mass and low muscle strength (24). On the other hand, sarcopenia, as defined in the healthy population, may not apply to dialysis population. Therefore, more research is needed in the future to determine the appropriate diagnostic cut-point values for sarcopenia in the dialysis population.

Consistently, previous studies found that malnutrition was significantly associated with sarcopenia in patients with MHD (26). With regard to the indicators used for nutritional assessment, studies have shown that the GNRI (27) and MIS (10) were associated with sarcopenia in patients with MHD. However, the relationship between the Cr index and sarcopenia remains scarce. Furthermore, the GNRI has the best discrimination to predict sarcopenia, while the Cr index similar to the MIS. However, Beberashvili et al. (28) reported that MIS is more comprehensive than GNRI in monitoring of nutritional status in patients with MHD. In addition, another study found that lower GNRI and Cr index values were both independently and equally associated with an increased risk of all-cause mortality in a multivariable-adjusted model (13). Differences of sample size, dialysis vintage, or population characteristics could explain this inconsistent finding. Our findings and hypotheses warrant further confirmation.

In our study, the cut-off value for estimating GNRI for sarcopenia was 103. In a previous study of Japanese dialysis patients, the cut-off value for GNRI was 90 when the outcome was mortality (19). The cut-off value in our study for sarcopenia was slightly higher, possibly due to the lower prevalence of malnutrition among patients in this population. The reason may be that universal health insurance system in Shanghai of China allows all the dialysis patients to treat complications early, and it should be recognized that many patients may be not malnourished due to their relative financial wealth. In addition, several research on the effectiveness of the GNRI in patients with MHD have shown that the GNRI is more sensitive and specific than other assessment tools in predicting mortality (29). Nutritional screening tool should be simple, fast, and reproducible (intra-rater and interrater reliability) and sufficiently discriminating. MIS is time-consuming and cumbersome because it requires a subjective evaluation. Moreover, GNRI requires only two parameters while the Cr index needs four parameters, making GNRI easier. The simpler of the two would be more practical and useful in the clinical setting, which is consistent with our results. Hence, GNRI may be used as a screening indicator based on medical record information in place of complex diagnostic criteria described in the consensus for estimating sarcopenia in patients with MHD.

This study has important implications for clinical practice and future research. First, it highlights that screening poor nutrition status is usually highly predictive of sarcopenia in patients with MHD. Second, it is strongly recommended to maintain sufficient calorie and protein intake especially physical therapy to avoid or reverse sarcopenia (26). However, efforts to increase food intake in patients with MHD with sarcopenia often are unsuccessful because of the persistent anorexia arising from sarcopenia-related inflammatory status (30). Finally, physical therapy may increase appetite in the short term by improving the patient's mood and increase metabolic rate in the long term by activating muscle metabolism.

Some limitations should be considered in our study. First, it is not possible to elucidate clear causal associations between three nutritional screening tools and sarcopenia in the patients with MHD because this study is a cross-sectional design. Second, the prevalence of malnutrition in this population is relatively low and few patients had severe malnutrition. Future studies with longitudinal designs are required to confirm the causal association between different nutritional screening tools and sarcopenia.

In conclusion, MIS, GNRI, and Cr indexes were almost equally and significantly associated with an increased risk of dynapenia, pre-sarcopenia, and sarcopenia. The sarcopenia predictability of the GNRI was greater than the Cr index and MIS, while the Cr index similar to MIS. Our findings provide further evidence for the selection of the nutritional screening tool to predict sarcopenia in patients with MHD. If further confirmed, GNRI as a simple tool could be used to identify patients with MHD at high risk of sarcopenia. Multiple interventions targeting these high-risk patients and implementing early intensification can help reduce their risk of sarcopenia and other clinical complications in the future.

## Data availability statement

The original contributions presented in the study are included in the article/supplementary material, further inquiries can be directed to the corresponding author.

## Ethics statement

The studies involving human participants were reviewed and approved by the Ethics Committee of Shanghai University of Medicine and Health Sciences. The patients/participants provided their written informed consent to participate in this study.

## Author contributions

XC, PH, and QG: study concept and design. PS, YZ, HZ, CY, JN, WD, and JZ: acquisition, analysis, and interpretation of data. XC and PH: drafting of the work. XZ, LZ, HQ, SZ, and QG: critical revision of the manuscript. All the authors contributed to the article and approved the submitted version.

## Funding

This work was supported by the funding of the National Natural Science Foundation of China (82172552), Shanghai Sailing Program (22YF1417900), the Clinical Trial Incubation Program From Tongji Hospital of Tongji University [ITJ(ZD)1808], and the Minhang District Medical Characteristic Specialty Construction Project (2020MWTZA01).

## Conflict of interest

The authors declare that the research was conducted in the absence of any commercial or financial relationships that could be construed as a potential conflict of interest.

## Publisher's note

All claims expressed in this article are solely those of the authors and do not necessarily represent those of their affiliated organizations, or those of the publisher, the editors and the reviewers. Any product that may be evaluated in this article, or claim that may be made by its manufacturer, is not guaranteed or endorsed by the publisher.

## References

[1] Chen LK Woo J Assantachai P Auyeung TW Chou MY Iijima K. Asian working group for sarcopenia: 2019 consensus update on sarcopenia diagnosis and treatment. J Am Med Dir Assoc. (2020) 21:300–7. 10.1016/j.jamda.2019.12.012 32033882

[2] Cruz-Jentoft AJ Baeyens JP Bauer JM Boirie Y Cederholm T Landi F. Sarcopenia: European consensus on definition and diagnosis: report of the european working group on sarcopenia in older people. Age Ageing. (2010) 39:412–23. 10.1093/ageing/afq034 20392703 PMC2886201

[3] Kim JK Kim SG Oh JE Lee YK Noh JW Kim HJ. Impact of sarcopenia on long-term mortality and cardiovascular events in patients undergoing hemodialysis. Korean J Intern Med. (2019) 34:599–607. 10.3904/kjim.2017.083 29161801 PMC6506738

[4] Macedo C Amaral TF Rodrigues J Santin F Avesani CM. Malnutrition and sarcopenia combined increases the risk for mortality in older adults on hemodialysis. Front Nutr. (2021) 8:721941. 10.3389/fnut.2021.721941 34604279 PMC8484646

[5] Mori K Nishide K Okuno S Shoji T Emoto M Tsuda A. Impact of diabetes on sarcopenia and mortality in patients undergoing hemodialysis. BMC Nephrol. (2019) 20:105. 10.1186/s12882-019-1271-8 30922266 PMC6437886

[6] Isoyama N Qureshi AR Avesani CM Lindholm B Barany P Heimburger O. Comparative associations of muscle mass and muscle strength with mortality in dialysis patients. Clin J Am Soc Nephrol. (2014) 9:1720–8. 10.2215/CJN.10261013 25074839 PMC4186520

[7] Carrero JJ Johansen KL Lindholm B Stenvinkel P Cuppari L Avesani CM. Screening for muscle wasting and dysfunction in patients with chronic kidney disease. Kidney Int. (2016) 90:53–66. 10.1016/j.kint.2016.02.025 27157695

[8] Manini TM Clark BC. Dynapenia and aging: an update. J Gerontol A Biol Sci Med Sci. (2012) 67:28–40. 10.1093/gerona/glr010 21444359 PMC3260480

[9] Kim JC Kalantar-Zadeh K Kopple JD. Frailty and protein-energy wasting in elderly patients with end stage kidney disease. J Am Soc Nephrol. (2013) 24:337–51. 10.1681/ASN.2012010047 23264684

[10] Vettoretti S Caldiroli L Armelloni S Ferrari C Cesari M Messa P. Sarcopenia is associated with malnutrition but not with systemic inflammation in older persons with advanced CKD. Nutrients. (2019) 11:1378. 10.3390/nu11061378 31248132 PMC6628018

[11] Roij DE Van Zuijdewijn CL Ter Wee PM Chapdelaine I Bots ML Blankestijn PJ. A Comparison of 8 nutrition-related tests to predict mortality in hemodialysis patients. J Ren Nutr. (2015) 25:412–9. 10.1053/j.jrn.2015.02.005 25820178

[12] Kalantar-Zadeh K Kopple JD Block G Humphreys MHA. malnutrition-inflammation score is correlated with morbidity and mortality in maintenance hemodialysis patients. Am J Kidney Dis. (2001) 38:1251–63. 10.1053/ajkd.2001.29222 11728958

[13] Yamada S Yamamoto S Fukuma S Nakano T Tsuruya K Inaba M. Geriatric nutritional risk index (GNRI) and creatinine index equally predict the risk of mortality in hemodialysis patients: J-DOPPS. Sci Rep. (2020) 10:5756. 10.1038/s41598-020-62720-6 32238848 PMC7113241

[14] Rasheedy D El-Kawaly WH. The accuracy of the geriatric nutritional risk index in detecting frailty and sarcopenia in hospitalized older adults. Aging Clin Exp Res. (2020) 32:2469–77. 10.1007/s40520-020-01492-5 32036578

[15] Kosoku A Uchida J Nishide S Kabei K Shimada H Iwai T. Association of sarcopenia with phase angle and body mass index in kidney transplant recipients. Sci Rep. (2020) 10:266. 10.1038/s41598-019-57195-z 31937826 PMC6959331

[16] Bassett DR. International physical activity questionnaire: 12-country reliability and validity. Med Sci Sports Exerc. (2003) 35:1396. 10.1249/01.MSS.0000078923.96621.1D 12900695

[17] Liu H Song B Jin J Liu Y Wen X Cheng S. Length of stay, hospital costs and mortality associated with comorbidity according to the charlson comorbidity index in immobile patients after ischemic stroke in China: a national study. Int J Health Policy Manag. (2022) 11:1780–7. 10.34172/ijhpm.2021.79 34380205 PMC9808248

[18] Marcelli D Usvyat LA Kotanko P Bayh I Canaud B Etter M. Body composition and survival in dialysis patients: results from an international cohort study. Clin J Am Soc Nephrol. (2015) 10:1192–200. 10.2215/CJN.08550814 25901091 PMC4491292

[19] Kobayashi I Ishimura E Kato Y Okuno S Yamamoto T Yamakawa T. Geriatric nutritional risk index, a simplified nutritional screening index, is a significant predictor of mortality in chronic dialysis patients. Nephrol Dial Transplant. (2010) 25:3361–5. 10.1093/ndt/gfq211 20400447

[20] Takahashi H Inoue K Shimizu K Hiraga K Takahashi E Otaki K. Comparison of nutritional risk scores for predicting mortality in Japanese chronic hemodialysis patients. J Ren Nutr. (2017) 27:201–6. 10.1053/j.jrn.2016.12.005 28215493

[21] Yamada K Furuya R Takita T Maruyama Y Yamaguchi Y Ohkawa S. Simplified nutritional screening tools for patients on maintenance hemodialysis. Am J Clin Nutr. (2008) 87:106–13. 10.1093/ajcn/87.1.106 18175743

[22] Arase H Yamada S Yotsueda R Taniguchi M Yoshida H Tokumoto M. Modified creatinine index and risk for cardiovascular events and all-cause mortality in patients undergoing hemodialysis: the Q-Cohort study. Atherosclerosis. (2018) 275:115–23. 10.1016/j.atherosclerosis.2018.06.001 29890446

[23] Shu X Lin T Wang H Zhao Y Jiang T Peng X. Diagnosis, prevalence, and mortality of sarcopenia in dialysis patients: a systematic review and meta-analysis. J Cachexia Sarcopenia Muscle. (2022) 13:145–58. 10.1002/jcsm.12890 34989172 PMC8818609

[24] LamarcaFCarreroJJRodriguesJCBigognoFGFetterRLAvesaniCM. Prevalence of sarcopenia in elderly maintenance hemodialysis patients: the impact of different diagnostic criteria. J Nutr Health Aging. (2014) 18:710–7. 10.1007/s12603-014-0505-525226111

[25] Yoowannakul S Tangvoraphonkchai K Vongsanim S Mohamed A Davenport A. Differences in the prevalence of sarcopenia in haemodialysis patients: the effects of gender and ethnicity. J Hum Nutr Diet. (2018) 31:689–96. 10.1111/jhn.12555 29611250

[26] Inaba M Okuno S Ohno Y. Importance of considering malnutrition and sarcopenia in order to improve the QOL of elderly hemodialysis patients in Japan in the Era of 100-year life. Nutrients. (2021) 13:2377. 10.3390/nu13072377 34371887 PMC8308469

[27] Lee H Kim K Ahn J Lee DR Lee JH Hwang SD. Association of nutritional status with osteoporosis, sarcopenia, and cognitive impairment in patients on hemodialysis. Asia Pac J Clin Nutr. (2020) 29:712–23. 10.6133/apjcn.202012_29(4).0006 33377365

[28] Beberashvili I Azar A Sinuani I Kadoshi H Shapiro G Feldman L. Comparison analysis of nutritional scores for serial monitoring of nutritional status in hemodialysis patients. Clin J Am Soc Nephrol. (2013) 8:443–51. 10.2215/CJN.04980512 23411424 PMC3586967

[29] Beberashvili I Azar A Sinuani I Shapiro G Feldman L Sandbank J. Geriatric nutritional risk index, muscle function, quality of life and clinical outcome in hemodialysis patients. Clin Nutr. (2016) 35:1522–9. 10.1016/j.clnu.2016.04.010 27117682

[30] Pourhassan M Babel N Sieske L Westhoff TH Wirth R. Inflammatory cytokines and appetite in older hospitalized patients. Appetite. (2021) 166:105470. 10.1016/j.appet.2021.105470 34139296

